# MesopTroph, a database of trophic parameters to study interactions in mesopelagic food webs

**DOI:** 10.1038/s41597-022-01831-3

**Published:** 2022-11-21

**Authors:** Mónica A. Silva, Catarina T. Fonseca, M. Pilar Olivar, Ainhoa Bernal, Jérôme Spitz, Tiphaine Chouvelon, Sigrún Jonasdottir, Ana Colaço, Vanda Carmo, Tracey Sutton, Gui Menezes, Tone Falkenhaug, Odd Aksel Bergstad, Sergi Pérez-Jorge

**Affiliations:** 1 Institute of Marine Sciences – OKEANOS & Institute of Marine Research – IMAR, University of the Azores, Rua Professor Doutor Frederico Machado 4, 9901-862 Horta, Portugal; 2 Institut de Ciències del Mar – Consejo Superior de Investigaciones Científicas (ICM-CSIC), Passeig Marìtim de la Barceloneta 37–49, 08003 Barcelona, Spain; 3 Centre d’Études Biologiques de Chizé (CEBC), UMR 7372 CNRS - La Rochelle Université, 405 Route de Prissé la Charrière, 79360 Villiers-en-Bois, France; 4 Observatoire Pelagis, UAR 3462 CNRS - La Rochelle Université, 5 Allées de l’Océan, 17000 La Rochelle, France; 5 Ifremer, Unité Contamination Chimique des Écosystèmes Marins (CCEM), Centre Atlantique, Rue de l’île d’Yeu, BP 21105, 44311 Nantes, France; 6 Technical University of Denmark, DTU Aqua, Kemitorvet, Building 202, 2800 Kgs Lyngby, Denmark; 7 Regional Directorate for Sea Affairs, Regional Secretariat for the Sea and Fisheries, Rua D. Pedro IV, 9900-111 Horta, Portugal; 8 Guy Harvey Oceanographic Research Center, Halmos College of Arts and Sciences, Nova Southeastern University, 8000 North Ocean Drive, Dania Beach, USA; 9 Institute of Marine Research, Flødevigen Research Station, 4817 His, Norway; 10 Nedre Brattbakken, 19 N-4835 Kristiansand, Norway

**Keywords:** Macroecology, Marine biology, Ecological networks

## Abstract

Mesopelagic organisms play a crucial role in marine food webs, channelling energy across the predator-prey network and connecting depth strata through their diel vertical migrations. The information available to assess mesopelagic feeding interactions and energy transfer has increased substantially in recent years, owing to the growing interest and research activity in the mesopelagic realm. However, such data have not been systematically collated and are difficult to access, hampering estimation of the contribution of mesopelagic organisms to marine ecosystems. Here we present MesopTroph, a georeferenced database of diet, trophic markers, and energy content of mesopelagic and other marine taxa compiled from 203 published and non-published sources. MesopTroph currently includes data on stomach contents, carbon and nitrogen stable isotopes, major and trace elements, energy density, fatty acids, trophic positions, and diet proportion estimates for 498 species/genera. MesopTroph will be expanded with new data emerging from ongoing studies. MesopTroph provides a unique tool to investigate trophic interactions and energy flow mediated by mesopelagic organisms, and to evaluate the ecosystem services of this community.

## Abstract (summary)

**Table:** 

Measurement(s)	Gastric Content • stable isotope analysis • major and trace elements • energy density • Fatty Acid • trophic position • Diet
Technology Type(s)	digital curation • digital curation
Sample Characteristic - Organism	Actinopteri • Mammalia • Aves • Malacostraca • Elasmobranchii • Cephalopoda • Copepoda • Reptilia • marine biota
Sample Characteristic - Environment	marine biome
Sample Characteristic - Location	North Atlantic Ocean • Mediterranean Sea

## Background & Summary

The ocean’s mesopelagic zone (200–1000 m depth) harbours highly diverse and abundant communities of fishes, cephalopods, crustaceans and gelatinous animals, particularly during daylight hours^1–3^. Collectively, the mesopelagic community displays a wide array of feeding behaviours and occupies a variety of trophic guilds, including organisms feeding on bacteria, detritus, phytoplankton, zooplankton, micronekton, and nekton^4–10^. In turn, mesopelagic organisms are the primary food source for many open-ocean predators (e.g., sharks, marine mammals, seabirds and marine turtles), and commercially harvested fishes and squids^11–16^. Additionally, many mesopelagic animals undergo diel vertical migrations, rising towards the surface at night to feed and returning to deeper waters during the day^17,18^. This daily migration represents an important mechanism for transferring surface primary production to deep-sea food webs and for the exchange of energy and organic matter between ocean layers^17,19,20^.

Despite their perceived importance, trophic pathways involving mesopelagic organisms are inadequately understood and their contribution to food web structure remains difficult to assess^21^. Identification of mesopelagic pathways has been hampered by the lack of quantitative data on feeding interactions between species or functional groups. Providing a comprehensive view of the mesopelagic food web is challenging due to the huge diversity of species and the inherent difficulties in sampling them. However, the data already available are extensive, as decades of research produced abundant information on stomach contents and various biochemical tracers (e.g., stable isotopes, fatty acids) for a broad range of marine taxa and regions.

To harness the potential of existing data, it would be necessary to assemble and standardize them, and make them readily accessible for the scientific community. Existing online diet databases of marine taxa (e.g., Fishbase^22^, SeaLifeBase^23^, DAPSTOM^24^, Food Habits Database^25^) are mostly limited to stomach contents and contain few records of mesopelagic organisms, while databases on trophic markers are taxonomically or geographically restricted (e.g., MarTurtSI^26^, SCAR^27^). Thus, large volumes of relevant data remain scattered in the literature or unpublished, making it difficult and laborious to find and reuse the data.

To address this gap, project SUMMER (https://summerh2020.eu/) created MesopTroph, a georeferenced database of diet, trophic biogeochemical markers, and energy content of marine taxa compiled from published and non-published sources, aimed to support research on mesopelagic food webs. MesopTroph comprises seven datasets: (i) diet compositions from stomach content analysis, (ii) stable isotopes (δ^13^C and δ^15^N), (iii) major and trace elements, (iv) energy density, (v) fatty acid trophic markers (FATM), (vi) trophic positions, and (vii) estimates of diet proportions (Tables 1 and 2).

**Table 1:** Trophic parameters in the data categories: Stomach contents, Stable isotopes, Major and trace elements, and Energy density.

Data category	Parameter short name	Description	Units
Stomach contents	N prey	Number of individuals of a prey type in the stomach of sampled individual(s)	
	N prey (prey no./total no. of prey)	Number of individuals of a prey type as a proportion of the total number of prey in the stomach of sampled individual(s)	%
	Occur (of prey type)	Number of times a prey type occurs in the stomachs of sampled individuals	
	Occur (percentage of predators containing a prey type)	Proportion of stomachs of sampled individuals containing a prey type	%
	Prey m	Weight of individuals of a prey type in the stomach of sampled individual(s)	g
	Prey m (weight of a prey type/total prey weight)	Weight of individuals of a prey type as a proportion of the total weight of prey in the stomach of sampled individual(s)	%
Stable isotopes	δ^15^N	Nitrogen isotope ratio	‰ air
	δ^13^C	Carbon isotope ratio: untreated samples, delipidized samples, lipid effect mathematically corrected	‰ PDB
	C/N	Ratio of total organic carbon to nitrogen: untreated samples, delipidized samples, lipid effect mathematically corrected	
Major and trace elements	Moisture	Percent moisture in the sample	%
	Ag, Al, As, B, Ba, Ca, Cd, Co, Cr, Cs, Cu, F, Fe, Hg, MeHg, MeHg(%), I, K, Li, Mg, Mn, Mo, Na, Ni, P, Pb, Sb, Se, Sr, V, Zn	Concentration of each trace metal	mg kg^−1^
Energy density	Energy density dm	Energy density per dry mass of the sample	kJ g^−1^
	Energy density wm	Energy density per wet mass of the sample	kJ g^−1^
	Moisture	Percent moisture in the sample	%

The parameter short name indicates the short form of the parameter name as defined in PANGAEA.

**Table 2:** Trophic parameters in the data categories: FATM, Trophic positions, and Estimates of diet proportions.

Data category	Parameter short name	Description	Units
FATM	Wet m	Wet mass of the sample	g
	Lipids/wm tot	Lipid content of the sample expressed in percentage of total wet mass	%
	C10:0, C12:0, C14:0, 14:1(n-5), C15:0, C16:0, 16:1(n-9), 16:1(n-7) (IUPAC: C16:1w7c), 16:1(n-5), 16:2, 16:2(n-6), 16:2(n-4), 16:2(n-3), 16:3, 16:3(n-4), 16:3(n-3), 16:4(n-1) %, 16:4(n-4), C17:0, 17:1(n-8), 17:1(n-7), C18:0, 18:1, 18:1(n-11), 18:1(n-9) (IUPAC: C18:1w9), 18:1(n-7) (IUPAC: C18:1w7), 18:1(n-5), 18:2(n-6), 18:2(n-4), 18:2(n-3), 18:3(n-6), 18:3(n-3), 18:4(n-4), 18:4(n-3), 18:5(n-3), 20:0, 20:1(n-11), 20:1(n-9), 20:1(n-7), 20:2(n-6), 20:3(n-6), 20:3(n-3), 20:4(n-6), 20:4(n-3), 20:5(n-3), 21:5(n-3), 22:0, 22:1(n-11), 22:1(n-9), 22:1(n-7), 22:3(n-3), 22:4(n-6), 22:5(n-6), 22:5(n-3), 22:6(n-3), 24:0, 24:1(n-9)	Concentration of each fatty acid in percentage of total fatty acids	%
Trophic positions	Trophic level	Trophic position of sampled individual(s) from stomach content or stable isotope analyses	
Estimates_Diet proportions	Diet p	Mean relative contribution of a prey type to the diet of sampled individuals	%

The parameter short name indicates the short form of the parameter name as defined in PANGAEA.

MesopTroph currently contains 5,149 data records assembled from 203 data sources, representing 72,821 specimens, 498 unique species or genera, 190 families, 17 classes, and eight phyla. While records encompass a wide range of taxonomic groups, most are from fishes (54%), mammals (18%), seabirds (10%), and malacostracan crustaceans (7%). MesopTroph covers all marine biogeographic realms identified in the eastern and central North Atlantic, and the Mediterranean^28^. Records date back to 1885 but most information is from 1980’s onwards. MesopTroph is far from complete but we plan to expand it in the future as more sources are identified and added, and as new trophic data emerges from the SUMMER project.

The database can be used to estimate the diet, trophic position, and consumption rates of individual taxa, and to model trophic interactions and energy flow through mesopelagic food webs. Such information is critical for investigating food web structure and inferring ecosystem functioning and resilience to fisheries and climate impacts. These data are also relevant to understand the role of mesopelagic organisms in carbon fluxes and sequestration. In conclusion, MesopTroph provides robust spatial and species coverage to support investigations into key ecosystem services provided by the mesopelagic community at a basin scale and help meet the growing demand for data for ecosystem-based management approaches.

## Methods

### Data sources

Data for the trophic parameters and data categories listed in Tables 1 and 2 were gathered from peer-reviewed scientific publications, grey literature (e.g., agency reports, theses, and dissertations) and unpublished data by the authors of this paper. Data compilation on stomach contents, stable isotopes, FATM, and trophic positions, focussed on mesopelagic organisms, their potential prey and predators. For major and trace elements, energy density and estimates of diet proportions, our search concentrated on mesopelagic taxa. Nevertheless, we also gathered information from small or intermediate-sized epi-, bathy- or benthopelagic species found in the compiled data sources. These species were included because they play key roles in most marine ecosystems, both as important consumers of phytoplankton and zooplankton, and prey for many top predators, and can represent alternative energy pathways to mesopelagic organisms. However, we stress that the data coverage for these species in the current version of the database is very incomplete. Our main interest was on data from the central and eastern North Atlantic, and the Mediterranean, corresponding to the study regions of the SUMMER project. When we could not find suitable data within this region, we extended the geographic scope of our literature search to the western North Atlantic. We did not search for datasets in open access repositories since those data can be easily accessed and extracted. However, some of the data provided by the authors of this paper have been previously deposited in PANGAEA.

DNA sequencing-based methods, such as metabarcoding and direct shotgun sequencing, are emerging as promising tools in dietary analyses due to the high resolution in taxonomic identification of many prey simultaneously, and the potential to provide quantitative diet estimates from relative read abundance^29^. However, recent studies have shown that various methodological and biological factors can break the correlation between the number and abundance of ingested prey and the prey DNA present in the sample, and lead to biased estimates of taxonomic diversity and composition of diet^29,30^. Given the uncertainties remaining in the interpretation of DNA sequencing-based diet data, we decided not to include these data in MesopTroph until additional research demonstrates that these techniques can be confidently applied for quantitative diet assessment.

We identified available data sources in the literature through systematic searches on *Web of Science*, *Google Scholar*, *ResearchGate*, and the *Google* search engine. We used multiple combinations of terms related to specific data categories (Table 3), in conjunction with the common or scientific taxon names (from genus to order), and the ocean basin. For example, the search for stomach content data of fishes belonging to the family Myctophidae was undertaken using the following terms: “stomach content” OR “gut content” OR “prey composition” OR “diet composition”, AND “mesopelagic fish” OR “myctophid” OR “Myctophiformes” OR “Myctophidae”, AND “Atlantic” or “Mediterranean”. For the mesopelagic and predator species known to be numerically abundant in the SUMMER study regions, we performed a second literature search using the common or scientific name of the species, along with the terms “diet”, “feeding habits”, “trophic ecology”, “trophic markers”, or “food web”. We also examined the literature cited within each collected publication to locate additional data sources.

**Table 3:** Terms used in the literature search for each data category.

Data category	Search terms
Stomach contents	stomach content OR gut content OR prey composition OR diet composition
Stable isotopes	stable isotopes OR isotopic composition OR nitrogen isotope OR carbon isotope
Major and trace elements	trace metal OR inorganic element OR macro-mineral OR micro-nutrient
Energy density	energy density OR energy content
FATM	fatty acid OR lipid composition
Trophic positions	trophic position OR trophic level
Estimates of diet proportions	diet proportion OR diet composition OR prey composition OR isotopic mixing model

We next screened the full text of the compiled studies and retained data sources that: (1) were collected within the region of interest, (2) reported quantitative data for the trophic parameters of interest, (3) reported the number of samples for pooled or aggregated data, and (4) provided sufficient details on the methodology to enable a quality check. In the case of stable isotope data, we only included data sources reporting both δ^13^C and δ^15^N measurements.

### Data extraction, cleaning, and formatting

We created a template table for each data category in Microsoft Excel to assemble all datasets into a single file, and to facilitate cleaning and standardization of data records. We added a large number of metadata fields to the tables to annotate details about the sampling (e.g., location, date, methods), sampled specimen(s) (e.g., taxonomy, number and size of individuals, number of replicates, tissue analysed), and data source (e.g., full reference, DOI) for every record.

Data contributors formatted and incorporated their datasets directly into the tables. For published sources, the data and associated metadata were extracted manually or digitized from the article text, tables, or supplementary material into the tables. Extraneous or hidden characters, and values such as “NA” (Not Available) or “ND” (Not Determined), were deleted from the parameter and metadata fields. Measurements of trophic parameters were standardized to the same units (see Tables 1 and 2). Parameter values that were clearly incorrect (e.g., δ^15^N > 20, or the frequency of occurrence of a prey higher than the number of stomachs sampled) were corrected by searching for the value within the data source. When values could not be corrected, we deleted that data record.

When available, we extracted information at the individual level. However, most studies reported data obtained from pooled samples of the same species. In some cases (e.g., small specimens such as planktonic organisms), a minimum and maximum number of individuals in the sample was provided instead of the actual number of individuals sampled. We added two columns to the tables presenting the minimum and maximum number of individuals in the sample. By filtering the column “Ind No (maximum per sample)” for values >1, users can easily identify records with aggregated data and differentiate them from records where information was drawn from a single individual (i.e., where “Ind No (maximum per sample)” =1). In addition, the tables Stomach contents and Estimates of diet proportions include a field “Sample ID” with a unique identifier of the sample. If data are reported at the individual level (i.e., “Ind No (maximum per sample)” =1) then Sample ID is the individual animal ID. If the data are from a group of individuals (i.e., “Ind No (maximum per sample)” >1), then Sample ID identifies that group.

We standardized the taxonomic classification and nomenclature of fishes and elasmobranchs following the Eschmeyer’s Catalog of Fishes (http://researcharchive.calacademy.org/research/ichthyology/catalog/fishcatmain.asp)^31,32^. For the remaining taxa, we used the World Register of Marine Species (http://www.marinespecies.org/)^33^. Unaccepted or alternate taxon names were replaced by the most up-to-date valid name. When the identification of a taxon was uncertain, the taxonomic level of identification was decreased to a satisfactory level. For example, prey reported as “Cephalopods” were changed to “Cephalopoda”, “Sepiolids” to “Sepiolidae”, and “*Myctophum punctatum*?” to the genus “*Myctophum*”.

### Stomach contents

Stomach contents analysis is a standard dietary assessment method that potentially enables quantifying diet components with high taxonomic resolution^34^. Three parameters are typically used to describe diet composition from stomach contents: the number of individuals of a prey type as a proportion of the total number of prey items (%N), the proportion of a prey item by weight or volume (%W), and the proportion of stomachs containing a particular prey item (i.e., percent frequency of occurrence, %F)^35^. When available, we collected data on the three parameters, as well as on the absolute number, weight, and frequency of occurrence of each prey type in the stomachs of each sampled individual or group of individuals. If stated in the data source, we indicate if prey weights were directly measured or reconstructed from hard remains (fish otoliths and vertebrae, cephalopod beaks), and if they represent dry or wet weight. Some datasets contained records of prey items without corresponding weights or numbers. As a result, the cumulative percent of all prey items did not sum to 100%. This occurred in 11 data records for the cumulative %W, and nine for the cumulative %N. While we checked the accuracy of percentage values and adjusted rounding errors, we did not attempt to fill in missing values nor did we remove records with missing values. When prey values were reported by an upper bound (e.g., “<0.01”), we assigned a value of half of the upper bound to that record and the percent of all prey items in that sample were rescaled to add to 100. Prey types were recorded at the highest taxonomic resolution.

### Stable isotopes

Bulk stable isotope ratios, primarily of carbon (δ^13^C) and nitrogen (δ^15^N), are increasingly used to examine predator-prey interactions and food web structure. Moreover, advances in isotopic mixing models allow the conversion of isotopic data into estimates of dietary contribution from different food sources^34,36^. We compiled δ^13^C and δ^15^N measured in specific tissues or in the whole body of individual organisms, or mean values (and standard deviations) for pooled samples. When available, the total organic carbon to nitrogen ratio (C/N) was also collected. Because lipids have more negative δ^13^C values relative to other major biochemical compounds in plant and animal tissues^37^, many studies correct for the lipid effect by extracting lipids from samples before analysis, or *a posteriori*, through mathematical corrections^38^. We searched the original data source to understand how the lipid effect was handled and separately report δ^13^C and C/N values for untreated and delipidized samples, as well as values that were mathematically-corrected.

### Major and trace elements

Organisms accumulate major and trace elements (including metals) directly from the external environment and/or indirectly through diet. As such, their elemental composition can help to infer dietary preferences, solve trophic links, and inform quantitative dietary analysis primarily based on stable isotopes or fatty acids^39,40^. We entered the individual concentrations (or mean concentrations, for samples with more than one individual) of all elements reported in a study into separate columns. We also collected the moisture content of the samples analysed (expressed in percentage). A column indicates whether concentrations are expressed on a dry mass (weight of the animal tissue after being dried) or wet mass (weight of the animal tissue containing water) basis, to allow the conversion between both types of data if necessary (using the moisture percentage).

### Energy density

Information on the energy density of prey is critical for estimating food requirements and consumption by predators, and modelling energy flux through food webs^41^. We collected data from studies that measured energy density directly by bomb calorimetry methods, and from studies that measured the proximate composition (i.e., the percentage of proteins, lipids and carbohydrates) of sampled tissues and converted these percentages into energy using combustion equivalents reported in the literature. When available, we collected energy density (or mean density, for samples with more than one individual) as a function of both dry and wet mass. When reported, the moisture percentage of samples was included to enable converting energy density between dry and wet mass.

### Fatty acid trophic markers (FATM)

Fatty acid (FA) analyses have been long used to qualitatively describe resource use, by tracing distinctive FA signatures of organisms into the lipids of consumers. More recently, quantitative fatty acid signature analysis and Bayesian models are emerging as powerful techniques to estimate the proportional contribution of different resources to consumer’s diet^34^. The FATM table compiles the proportion of each FA measured in sampled tissues or in the whole body of organisms in relation to total FAs analysed. For pooled samples, we collected the mean and standard deviation (when available) of the percentage FA. In all data records, we included the full range of FAs with values above 0.1% but excluded FAs that were measured together. Care should be taken, however, since these percentages are not absolute but are affected by the number and particular mix of FAs analysed in each study^42^.

### Trophic positions

Trophic position (TP) is a continuous measure of the position of a species in the flow of energy from the bottom to the top of a food web, and estimation of TP is fundamental to the analysis of food webs^43^. TP has traditionally been estimated using the relative contribution of prey to a consumer’s diet based on stomach content analysis, but isotope analysis (e.g., bulk tissue or compound-specific) is increasingly used to estimate TP, based on the progressive enrichment in heavy isotopes (mainly ^15^N) from preys to consumers^38^. We collected TP derived both from stomach content and stable isotope analyses, specifying the type of analysis under the field “Method”. When reported in the original data source, we also present information on the type of models (e.g., additive model with constant isotopic enrichment (TPA), isotopic mixing model, mass-balanced trophic model), baseline taxa, and trophic enrichment factors used to estimate TP^44,45^.

### Estimates of diet proportions

Advances in analytical and statistical techniques now enable quantitative estimation of diet from biochemical tracers such as bulk or compound-specific stable isotopes, fatty acids, or trace elements. Linear or Bayesian mixing models use the biotracer composition (e.g., isotopic composition, fatty acid profiles) of a mixture (i.e., consumer) to estimate the relative proportions of the different sources (i.e., prey) in that mixture^36,46^. We collected diet proportions (i.e., the relative proportion of a prey item to the diet of a consumer) of small mesopelagic taxa derived from isotope mixing models. We focused on these taxa because their small and soft‐tissued prey are often difficult to identify and quantify through visual methods. In the future, we expect to add estimates of diet proportions from ongoing analysis of other biochemical tracers, and for a wider range of consumers. As such, the table includes columns to specify the type of analysis and models used under “Method” and “Method comment”, respectively.

### Metadata

For each data record, we retrieved the sampling location as provided in the source, and geographic coordinates. If coordinates were not reported, the midpoint coordinates of the study area or of data records was determined and appended to the data record. All geographic coordinates were converted to decimal degrees. We categorized the study locations into seven different regions: Mediterranean (when the location within the basin was not specified), Eastern Mediterranean, Western Mediterranean, Atlantic-tropical, Atlantic-temperate, Atlantic-subarctic, and Atlantic-Arctic. Most data sources did not provide the precise date for the collection of each sample, and usually data collection spanned over months or years; thus, the first and last months and years of the sampling were annotated. Some data sources did not provide sufficient details to complete all metadata fields, but we still extracted the trophic parameters. In the current version of MesopTroph, sampling location is missing for seven data records (0.1% of total), sampling year for 218 (4.2%), and sampling month for 748 (14.5%).

We added the phylum, class, order and family for every taxon. Finally, we categorized each taxon as “Mesopelagic”, “Non-mesopelagic” or “Unknown”. Mesopelagic taxa include fish, cephalopods, crustaceans, and gelatinous organisms inhabiting the mesopelagic zone (200–1000 m depth)^47–49^. Non-mesopelagic taxa encompass benthic and pelagic organisms that do not use the mesopelagic zone, as well as elasmobranchs, marine mammals, seabirds and marine turtles. Data records where the taxon was identified only to higher taxonomic ranks that include both mesopelagic and non-mesopelagic organisms were classified as Unknown.

## Data Records

**Fig. 1:**
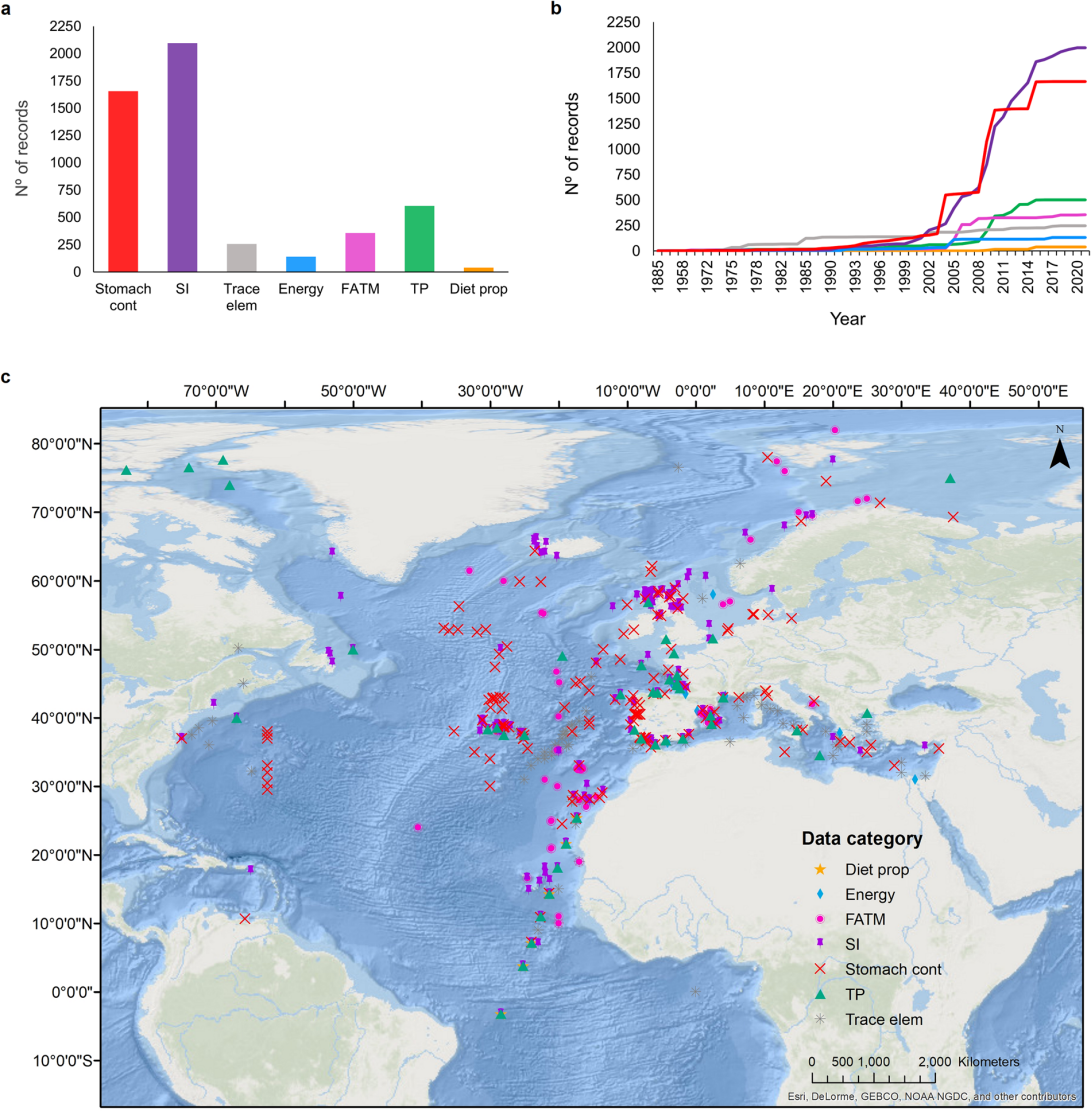
Data content of the MesopTroph database per data category. (**a**) Number, (**b**) cumulative temporal distribution, and (**c**) geographic distribution of data records for each data category. The time series displayed in panel b are based on 4,942 data records (96% of total records) with information on sampling year. Map in panel c shows unique sampling locations of 5,139 (99.9% of total) data records. Data categories are: Stomach contents (Stomach cont), Stable Isotopes (SI), Major and trace elements (Trace Elem), Energy density (Energy), Fatty acid trophic markers (FATM), Trophic positions (TP), and Estimates of diet proportions (Diet prop). Color of time series in panel b indicates data categories corresponding to panel a.

MesopTroph currently contains 1,655 diet data records from 16,396 stomach contents, 2,095 stable isotope signatures of δ^13^C and δ^15^N, 256 records of major and trace elements, 140 measurements of energy density, 359 records of FATM, 605 estimates of trophic positions, and 39 estimates of diet proportions (Fig. 1a). The majority of data records are from samples collected between 2000 and 2015 (Fig. 1b). Data records spread across the entire Mediterranean basin, and the Northeast Atlantic, from the European continent over to the Mid-Atlantic Ridge (with a few samples from the Northwest Atlantic and South Atlantic), but the geographic distribution is biased towards Atlantic temperate and tropical waters, and the Mediterranean. Data from stomach contents and stable isotopes are more evenly distributed within the study region, while estimates of diet proportions are only available for samples from the Mediterranean and tropical Atlantic, and energy density is only available for samples from the Mediterranean and temperate Atlantic (Fig. 1c).

**Fig. 2:**
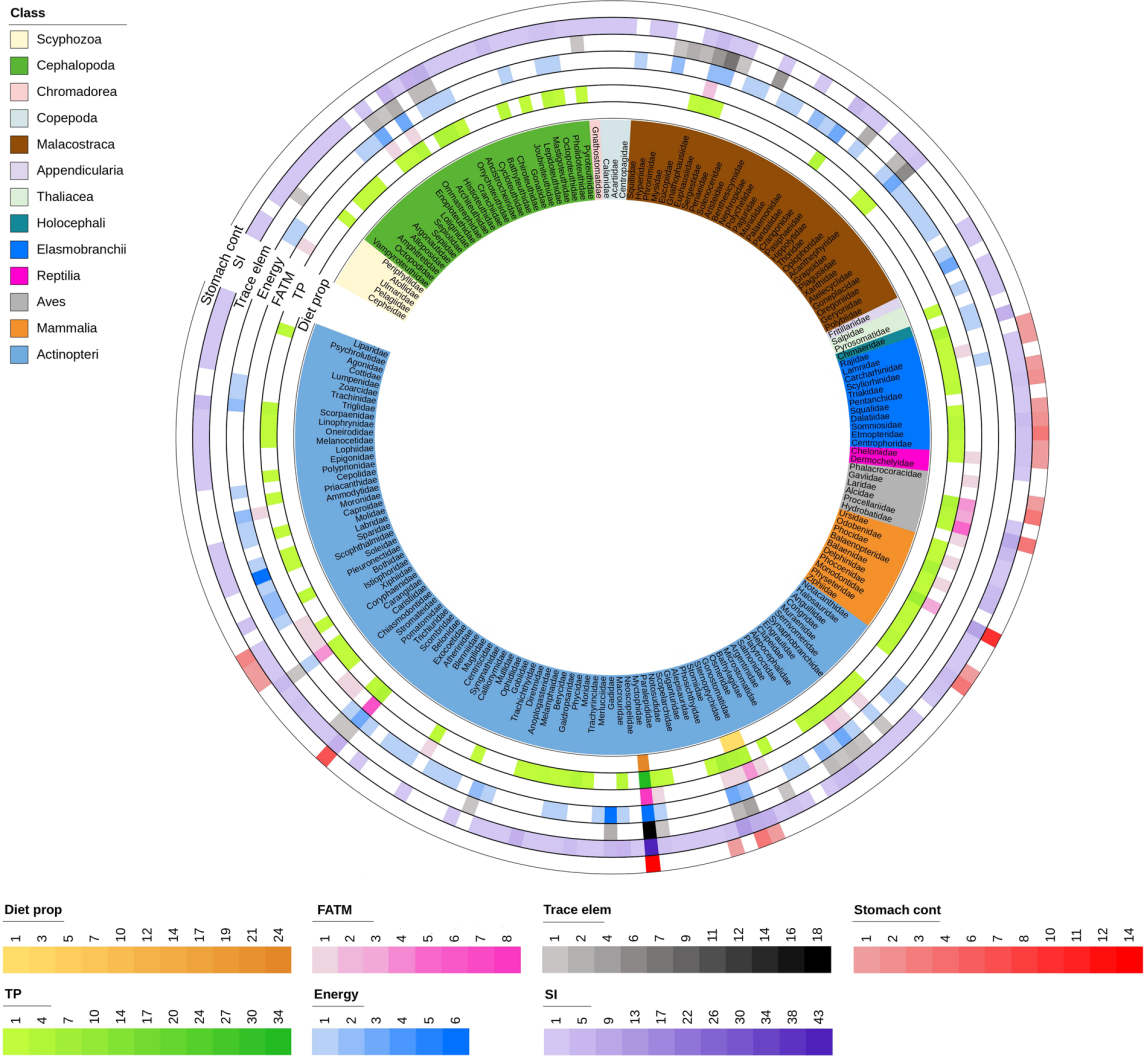
Number of unique species/genera per Family and data category in the MesopTroph database. The figure does not include 31 data records from organisms identified to taxonomic ranks higher than family. Data categories are: Stomach contents (Stomach cont), Stable Isotopes (SI), Major and trace elements (Trace Elem), Energy density (Energy), Fatty acid trophic markers (FATM), Trophic positions (TP), and Estimates of diet proportions (Diet prop).

**Fig. 3:**
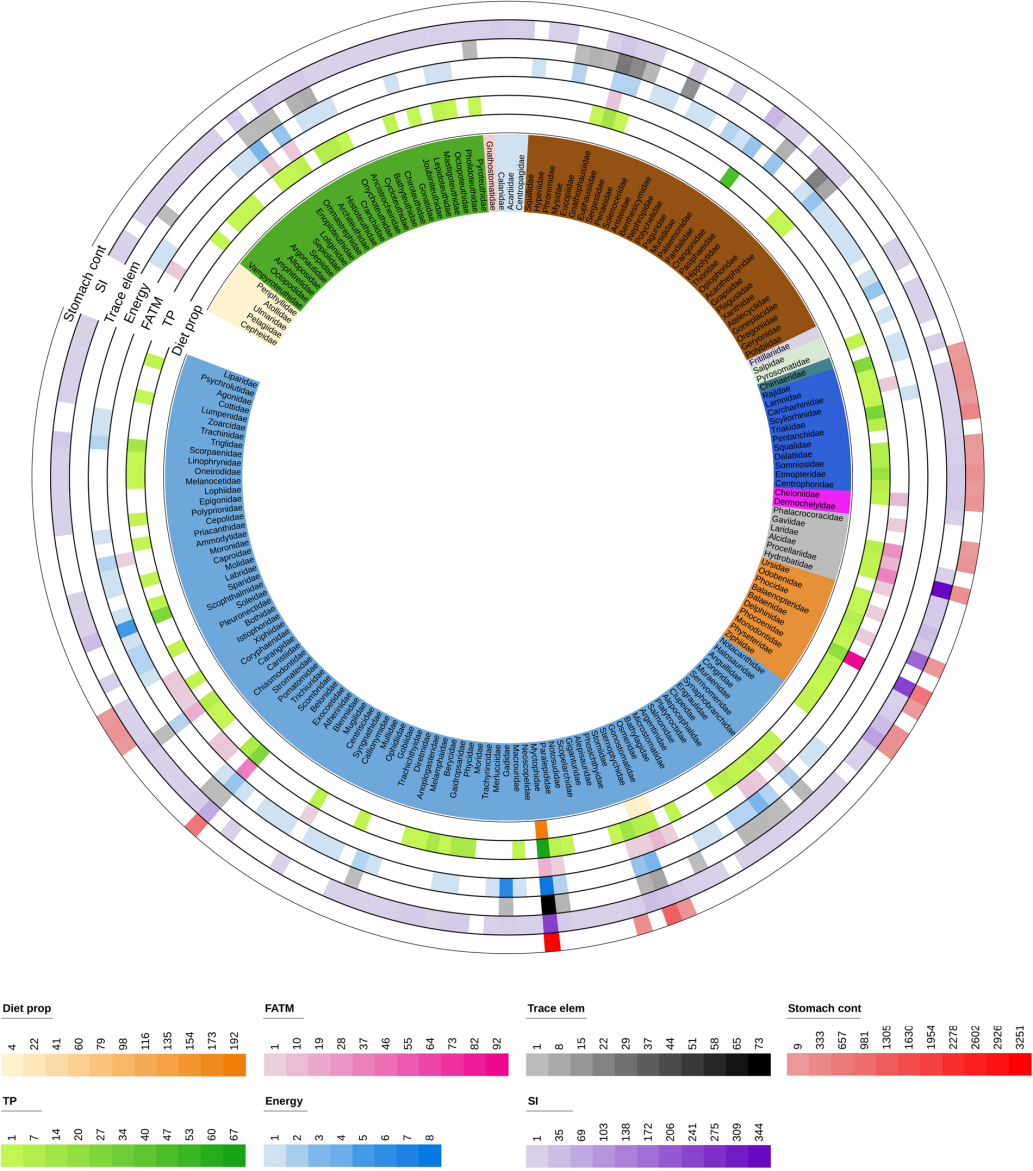
Number of data records per Family and data category in the MesopTroph database. The figure does not include 31 data records from organisms identified to taxonomic ranks higher than family. Data categories are: Stomach contents (Stomach cont), Stable Isotopes (SI), Major and trace elements (Trace Elem), Energy density (Energy), Fatty acid trophic markers (FATM), Trophic positions (TP), and Estimates of diet proportions (Diet prop).

Taxonomic coverage also varies among data categories. The Stable isotopes and Trophic positions tables include the highest diversity of taxonomic groups and species (Fig. 2). Stable isotope data are available for 348 unique species or genera, including 185 pelagic and benthic fishes, 47 cephalopods, 31 marine mammals, 30 crustaceans, 26 elasmobranchs, 16 seabirds, four marine turtles, four cnidarians, three copepods, and two tunicates (Fig. 2), in addition to several samples only identified to higher taxonomic levels. Nearly 33% of data records are from fishes, 31% from marine mammals, 17% from seabirds, and 6% from crustaceans (Fig. 3). The Trophic positions table contains data on 204 unique species/genera, of which 108 are fishes (accounting for 46% of data records), 30 cephalopods (6%), 25 marine mammals (7%), 19 elasmobranchs (17%), 10 seabirds (2%), 10 crustaceans (15%), one marine turtle (0.3%), and one tunicate (0.5%) (Figs. 2 and 3). Stomach content data are available for 20 mesopelagic fishes, which represent 85% of the data records in this table. The remaining records belong to top predators such as marine mammals (7%), elasmobranchs (3%), large pelagic fishes (3%), seabirds (<2%), and one marine turtle (<0.5%) (Figs. 2 and 3). Likewise, the FATM table includes data on mesopelagic fish, crustaceans, cephalopods, and cnidarians, as well as on their potential top and mesopredators. Energy density values are reported for 121 species or genera of fish, crustaceans, and cephalopods characteristic of meso- to bathypelagic waters, or living on the continental shelf, 96% of the data records in the table Major and trace elements are of mesopelagic organisms (fishes, crustaceans, and cephalopods), and estimates of diet proportions are provided only for the fish families Myctophidae, Sternoptychidae, and Gonostomatidae (Figs. 2 and 3).

In terms of the information available for different parameters within each data category, most data records in the Stomach contents table report either the absolute number (91%) or the percent number of prey (97%), whereas the absolute or percent weigh is reported in 24% and 32% of the records, respectively, and only 8% of records include the percent frequency of occurrence of prey. δ^15^N values are available for all the data records, 44% of data records report δ^13^C values for untreated samples, 43% report δ^13^C values for delipidized samples, and in 37% of the records δ^13^C values were mathematically corrected. C/N ratios are available for 30% of the records. The table Major and trace elements contains concentrations of 31 elements, but each data record includes on average six elements (range: 1–20). The most reported elements are Cadmium (66%), Zinc (63%), Copper (58%), and Iron (49%). Similarly, each data record in the FATM table include the proportion of 8–36 fatty acids (average of 19.4), the most common being palmitic, stearic, DHA, EPA, and oleic acids, available for >97% of records. Overall, 66% of all data records included in the current version of MesopTroph are drawn from single individuals but these percentages varied greatly across datasets (Stomach contents, 90%; FATM, 76%; Stable Isotopes, 71%; Trophic positions, 54%; Major and trace elements, 13%; Energy density and Estimates of diet proportions, 0%).

MesopTroph is available for download at the World Data Center PANGAEA. A parent dataset (10.1594/PANGAEA.946020^50^) merges seven datasets, that can be downloaded separately as tab-delimited files, or in a single ZIP file:

1. Stomach contents – stomach content records, presented as frequency of occurrence, numbers or weight of a food item (10.1594/PANGAEA.946139^51^);
2. Stable Isotopes – isotope ratios of carbon (δ^13^C) and nitrogen (δ^15^N) (10.1594/PANGAEA.945917^52^)
3. Major and trace elements – concentrations of essential and trace elements (10.1594/PANGAEA.945907^53^);
4. Energy density – estimates of energy density of total body mass of organisms determined by calorimetric methods (10.1594/PANGAEA.945870^54^);
5. FATM – fatty acid profiles, expressed as percent of total fatty acids (10.1594/PANGAEA.945881^55^);
6. Trophic positions – estimates of the trophic position of organisms calculated from the analyses of stomach contents or stable isotopes (10.1594/PANGAEA.945968^56^);
7. Estimates_Diet proportions – estimates of the mean proportional contribution (±standard deviation) of a food item to the diet of a sample of predators calculated from analyses of biochemical tracers (10.1594/PANGAEA.945874^57^);

The parent dataset (10.1594/PANGAEA.946020^50^) also provides access to a data dictionary, listing the fields in all datasets, along with their description, format, and units (when applicable).

The first row of each dataset contains the abbreviated name of the parameters, and the first column contains a unique record identifier (Record number). The datasets provide information on the location, dates and method of sample collection, taxonomic ranks (phylum, class, order, family), number and size (or size range) of sampled organisms, method/model used in data analysis. All published data records include the full citation and persistent identifier of the data source and data (when available). For unpublished data records, the name and affiliation of researchers that provided the data are given.

## Technical Validation

Most unpublished data included in MesopTroph were provided by authors of this paper, who are experts on the organisms and techniques used. Data extracted from the literature underwent a basic quality check by verifying the appropriateness of the sampling and analytical methods in the original publication. After entry into the database, several manual and automated checks were performed to ensure that the entered data were correct. This included checking for missing information, duplicate entries, typos, spelling errors or inconsistencies. Values of all trophic parameters were visually evaluated to detect values far outside the expected range for that parameter. Clear outliers were verified and corrected by comparing with the original data source. When the outlier was not the result of a transcription error and was as reported in the data source, we either deleted the value for that specific parameter, or removed the corresponding record. Scientific names and taxonomy were checked against the Eschmeyer’s Catalog of Fishes^31,32^ and WoRMS^33^ to ensure taxonomic consistency. Finally, all data records were verified by a reviewer and suspicious entries were re-checked against the original source.

## Usage Notes

MesopTroph is available for download from PANGAEA as a compressed folder containing the tab-delimited files for all data categories and a data dictionary text file, or as separate files. Data are released under the CC-BY licence, meaning that there are no restrictions on its use, but it should be appropriately referenced by citing the present paper. If a single or a few specific datasets are used separately from the database, we encourage users to cite the original data source in addition to MesopTroph database. The database will be updated in the next few years with data from the literature, and raw data and analytical outputs emerging from the SUMMER project. Expected additions include expanding the taxonomic and geographic coverage of the database, and increasing the sample size with data from recent years.

## Data Availability

No code was used in this study.
